# Dermatoscopic markers of disease activity in vitiligo

**DOI:** 10.1016/j.jdin.2025.04.017

**Published:** 2025-07-28

**Authors:** Saâdia Boughaleb, Meryem Soughi, Ghita Sqalli, Aida Oulehri, Marwa El Baldi, Zakia Douhi, Sara Elloudi, Hanane Baybay, Karima El Rhazi, Fatima Zahra Mernissi

**Affiliations:** a Dermatology and Venerology Department, Hassan II University Hospital, Fez, Morocco; b URL CNRST N15, Human Pathology, Biomedecine and Environment Laboratory, Faculty of Medicine, Pharmacy and Dental, Sidi Mohamed Ben Abdellah University, Fez, Morocco; c Laboratory of Epidemiology and Scientific Research, Faculty of Medicine, Pharmacy and Dental, Sidi Mohamed Ben Abdellah University, Fez, Morocco

**Keywords:** activity, dermoscopy, progressive, recent, repigmentingstable, vitiligo

## Abstract

**Background:**

Determining vitiligo activity is essential for prognosis and treatment planning.

**Objectives:**

To describe the dermoscopic findings and correlate them with the activity of the disease.

**Methods:**

A single-center, descriptive, analytical study included 233 patients (330 lesions). Lesions were categorized as progressing, repigmenting, stable, or recent. Dermoscopic analysis was performed using a DermLite 4 dermatoscope, with statistical testing through Pearson’s chi-square.

**Results:**

Progressing lesions were associated with trichrome pattern, reverse network, nacreous white globules, starburst, comet-tail pattern, micro-Koebner phenomenon, residual peripilar pigmentation with leukotrichia, perilesional polka dots, perifollicular depigmentation, and reverse network (*P* < .01). Stability was indicated by sharp borders (*P* < .01). Repigmenting lesions exhibited perifollicular or border hyperpigmentation, erythema, and telangiectasias (*P* < .01). Early vitiligo was suggested by hypopigmented lesions with attenuated network or perifollicular depigmentation (*P* < .01). Newly identified markers included distally pigmented bicolored hair for recent lesions and proximally pigmented bicolored hair for repigmentation.

**Limitations:**

Most patients had prior treatment, potentially influencing dermoscopic patterns.

**Conclusion:**

This study, based on a large sample size, identifies specific dermoscopic markers for vitiligo activity and introduces novel findings like bicolored hairs. These insights can refine clinical assessment and serve as a foundation for future research.

**Capsule Summary**

• This study confirms known dermoscopic markers of vitiligo activity and, through analysis of a large sample, identifies novel indicators such as bicolored hairs.
• Standardized nomenclature and a systematic approach, center/border/periphery, are proposed to enhance dermoscopic analysis in vitiligo.

## Introduction and objectives

Vitiligo is an autoimmune disorder characterized by the loss of skin pigmentation, with no predilection for sex, age, or ethnicity. Recent advances in understanding its pathogenesis have opened new therapeutic perspectives.^1^ Clinically, vitiligo presents as hypopigmented or achromic macules of varying size and shape, often symmetrical and, in some cases, segmental. The course of vitiligo is typically recurrent, influenced by triggering factors or relapses mediated by resident memory T cells.^2^

Accurately determining disease activity is crucial for predicting repigmentation potential and guiding therapeutic decisions. While certain clinical features have been identified as reliable activity markers,^3^ dermoscopy has proven invaluable in differentiating vitiligo from other causes of acquired hypopigmentation. On dermoscopy, vitiligo lesions are characterized by depigmented areas with perifollicular pigment reservoirs—a feature not observed in other depigmentation disorders.^4^

Few studies have statistically correlated dermoscopic features with disease activity.^5^ However, findings in the literature have been inconsistent and sometimes contradictory due to study design limitations, reliance on patient history, and the lack of standardized dermoscopic definitions.^6^ Consequently, the need for standardized assessments and prospective studies has been emphasized by authors to address these limitations.^7^

The aim of this study is threefold: to comprehensively review dermoscopic signs associated with vitiligo, to determine their statistical correlation with disease activity, and to propose a standardized algorithm and nomenclature to simplify dermoscopic evaluation of vitiligo lesions.

## Material and method

### Study design

We conducted a prospective, transversal, single-center, descriptive, and analytical study over 2 years at the Dermatology Department of Hassan II University Hospital. According to the lesion stability theory, the same patient can have lesions at distinct evolutionary stages.^8^^,^^9^

A photographic database of vitiligo lesions was created for patients treated in the department. 2 clinicians categorized the lesions into 4 groups based on clinical evolution and previous patient photographs.

• *Progressing:* Lesions that appeared and/or expanded within the past year.
• *Repigmenting:* Lesions showing decreased size, darkening, or repigmentation over the past year, with or without treatment.
• *Stable:* Lesions that remained unchanged over the past year.
• *Recent onset lesions:* Hypopigmented lesions evolving for less than 3 months.

We collected clinical and Wood’s lamp images of each lesion. Dermoscopic images of the lesion’s center, border, and perilesional skin were captured using a DermLite4 dermoscope coupled to a smartphone.

### Dermoscopic features in vitiligo

Based on published literature, we performed a comprehensive review and standardized the description of dermoscopic signs (Table I). This step aimed to minimize interobserver variability.

**Table I:** Proposed definition of the dermoscopic signs in vitiligo

Dermoscopic sign	Definition	References
White structureless areas	Plain white area devoid of any pigment network	^10^
Reverse network	Network composed of depigmented white lines surrounding areas of relatively pigmented skin	
Attenuated network	Network with reduced pigmentation compared to the network of normal skin	
White nacreous globules	Lesion formed by multiple small, rounded achromic areas, > 1 mm in size, with a tendency to coalesce	Not reported
Starburst pattern	A rounded or oval white patch with peripheral projections, resembling a star	^5^
Comet-tail pattern	Oval whitish area with linear streaks that gradually thin out	
Trichrome pattern	A three-color gradient: an achromic center, a hypochromic intermediate zone, and a normal pigmented peripheral ring	
Micro-Koebner	Fine white linear striae due to mechanical Koebner phenomenon, often barely visible to the naked eye	^11^
Intralesional erythema	Red background within the vitiligo lesion	^6^
Telangiectasias	Linear and branched red vessels within the depigmented area	
Dotted vessels	Pinpoint red vessels (sub-millimeter) scattered within the depigmented area	Not reported
Leukotrichia	White hair resulting from the loss of pigmentation	^6^
Distally pigmented bicolored hair	Bicolored hair that has lost its pigmentation at emergence, with persistence of normal hair coloration at the end	Not reported
Proximally pigmented bicolored hair	Bicolored hair with persistence of color at emergence, and depigmentation of distal part	
Perifollicular residual pigment	Dot-like homogeneous < 1 mm pigmentation of the pilosebaceous follicle opening	^6^
Perifollicular hyperpigmentation	Homogenous pigmentation or pigmented network > 1 mm in diameter, of the pilosebaceous follicle opening	
Sharp border	Well-defined achromic zone	^11^^,^^12^
Blurred border	Poorly defined border giving unclear demarcation with the perilesional skin	
Hyperpigmented border	Focal or complete hyperpigmentation of the margin, between the depigmentation and the perilesional skin	^11^
Perilesional reverse network	Reverse network located at the periphery of a lesion, on a normal-appearing skin	
Perilesional white nacreous globules	Round depigmented globules located at the periphery of a lesion	
Polka dots	Multiple rounded white dots, well-defined and < 1 mm in size, located around the white area and completely invisible to the naked eye	^13^
Perilesional erythema	Red background surrounding the vitiligo lesion	^13^
Perilesional leukotrichia	White hairs surrounding the main lesion	^6^
Perilesional perifollicular depigmentation	Absence of pigment around the opening of the pilosebaceous follicle in normal-appearing skin at the periphery of lesions	^5^

### Dermoscopy and statistical analysis

**Fig 1:**
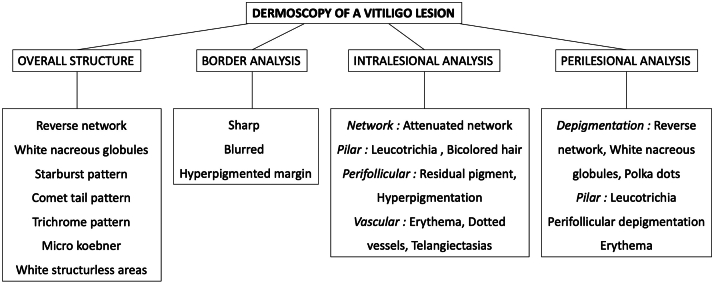
Dermoscopy analysis chart for vitiligo lesions.

The 2 clinicians analyzed each lesion following a proposed algorithm (Fig 1) to evaluate.

1. *General lesion structure:* Reverse network, white nacreous globules, starburst pattern, comet-tail pattern, trichrome areas, micro-Koebner phenomenon, and white structureless zones.
2. *Intralesional details:* Network, pilar/peripilar regions, and vascular patterns.
3. *Lesion border:* Sharp or blurred edges and presence of hyperpigmentation.
4. *Perilesional skin:* Types of depigmentation, pilar/peripilar findings, and vascular patterns.

Data were recorded using a standardized data sheet. Qualitative variables were expressed as percentages for each dermoscopic sign across the activity groups. Statistical analyses were performed using *SPSS software version 21*. Categorical data were analyzed using Pearson’s chi-square test, with *P < .05* considered statistically significant.

## Results

### Clinical profile

A total of 233 patients with vitiligo were included in the study, comprising 147 women (63%) and 86 men (37%). The mean age was 32.5 years, ranging from 2 to 85 years. Non-segmental vitiligo was the predominant type, accounting for 91% of cases.

In total, 330 dermoscopic images of vitiligo lesions were analyzed. Based on the lesions' evolutionary status.

− 37.6% were categorized as progressing.
− 22.7% were repigmenting.
− 20.3% were stable.
− 19.4% were recent onset lesions.

### Dermoscopic features in vitiligo

A detailed evaluation of 25 dermoscopic signs was performed for each lesion based on clear definitions, derived from both our observations and the literature, and summarized in Table I. To enhance reproducibility and minimize interobserver variability, overly metaphorical terms, such as “salt and pepper” (Wali et al), were avoided. Emphasis was placed on concise and standardized definitions.

Key dermoscopic features distinguishing vitiligo from other hypopigmentation conditions were identified:

- White structureless areas, often associated with residual pigmentation in the follicular region, a hallmark feature.^4^
- Network alterations, particularly reverse network and attenuated network patterns, previously described by Thatte et al^10^

Additional specific dermoscopic patterns highlighted by Jha et al^13^ were included:

- White nacreous globules (rounded achromic structures)
- Starburst appearance (radiating star-like projections)
- Comet-tail patterns (linear white streaks)
- Trichrome lesions, defined by a gradient of pigmentation transitioning from an achromic center to a hypochromic intermediate zone, surrounded by a pigmented periphery (Wali et al).^14^

Vascular signs such as erythema, dotted vessels, and telangiectasias were frequently noted, particularly in lesions undergoing treatment.^14^ Furthermore, perifollicular changes, such as residual pigment, hyperpigmentation, and depigmentation, have been comprehensively reviewed by Godínez-Chaparro et al.^5^

Finally, border characteristics were emphasized, with a clear distinction between sharp and blurred edges, as described by Nirmal et al.^12^ The algorithm for dermoscopic analysis (Fig 1) was systematically applied and ensured a robust assessment of each vitiligo lesion.

### Descriptive analysis

Key dermoscopic findings (detailed in Table II) include:

1. Overall lesion patterns
  • White structureless areas were the most frequent feature (77.9%).
  • Additional patterns included trichrome lesions (30.3%), reverse network (25.8%), white nacreous globules (22.4%), starburst (12.4%), comet-tail streaks (11.2%), and micro-Koebner phenomenon (12.4%).
2. Intralesional features
  • Network alterations were dominated by attenuated network (22.4%).
  • Follicular changes.
  • Leukotrichia (47.3%) was most common.
  • Novel findings included distally pigmented hairs (4.5%) and proximally pigmented hairs (0.9%).
  • Perifollicular pigmentation: Hyperpigmentation (30.3%) and smaller, homogenous residual pigment (18.5%).
  • Vascular patterns: Erythema (44.8%), dotted vessels (29.4%), and telangiectasias (24.8%).
3. Borders: Blurred in 74% and sharp in 26%, with hyperpigmentation in 31%.
4. Perilesional findings
  • Peripheral reverse network (33.6%), polka dots (12.7%), and white nacreous globules (23%).
  • Erythema (13.3%) and follicular changes such as leukotrichia (27%) and perifollicular depigmentation (17.6%).

**Table II:** Detail of the dermoscopic findings in each activity group

Dermoscopic Finding	Progressive (*N* = 124)	Stable (*N* = 67)	Repigmenting (*N* = 75)	Recent Onset (*N* = 64)	*P* value
Overall structure					
Reverse network	34.7 (43)	3.0 (2)	21.3 (16)	37.5 (24)	<.01∗
White nacreous globules	43.5 (54)	0	12.0 (9)	17.2 (11)	<.01∗
Starburst	25.8 (32)	1.5 (1)	2.7 (2)	9.4 (6)	<.01∗
Comet tail	26.6 (33)	0	1.3 (1)	4.7 (3)	<.01∗
Trichrome	51.6 (64)	4.5 (3)	25.3 (19)	21.9 (14)	<.01∗
Micro-Koebner	30.6 (38)	0	0	4.7 (3)	<.01∗
Borders					
Sharp	8.1 (10)	74.6 (50)	26.7 (20)	4.7 (3)	<.01∗
Blurred	91.9 (114)	25.4 (17)	73.3 (55)	95.3 (61)	<.01∗
Hyperpigmented	17.7 (22)	23.9 (16)	84.0 (63)	4.7 (3)	<.01∗
Intralesional					
Attenuated network	18.5 (23)	6.0 (4)	33.3 (25)	34.4 (22)	<.01∗
Erythema	42.7 (53)	31.3 (21)	73.3 (55)	29.7 (19)	<.01∗
Telangiectasias	29.0 (36)	11.9 (8)	48.0 (36)	3.1 (2)	<.01∗
Dotted vessels	42.7 (53)	20.9 (14)	28.1 (18)	28.1 (18)	<.01∗
Leukotrichia	53.2 (66)	34.3 (23)	48.0 (36)	48.4 (31)	.097
Distally pigmented bicolor hair	0.8 (1)	0	0	21.9 (14)	<.01∗
Proximally pigmented bicolor hair	0	0	4.0 (3)	0	.016∗
Perifollicular residual pigment	28.2 (35)	10.4 (7)	17.3 (13)	9.4 (6)	.003∗
Perifollicular hyperpigmentation	25.8 (32)	13.4 (9)	78.7 (59)	0	<.01∗
Perilesional					
Perilesional reverse network	49.2 (61)	4.5 (3)	33.3 (25)	34.4 (22)	<.01∗
Perilesional white nacreous globules	46.8 (58)	6.0 (4)	6.7 (5)	14.1 (9)	<.01∗
Polka dots	30.6 (38)	0	1.3 (1)	4.7 (3)	<.01∗
Perilesional erythema	17.7 (22)	1.5 (1)	25.3 (19)	3.1 (2)	<.01∗
Perilesional leukotrichia	33.1 (41)	11.9 (8)	21.3 (16)	37.5 (24)	.002∗
Perilesional perifollicular depigmentation	30.6 (38)	3.0 (2)	2.7 (2)	25.0 (16)	<.01∗

∗ Significant *P* value.

### Statistical analysis

Univariate analysis revealed statistically significant associations between dermoscopic findings and lesion activity groups (Table III).

**Fig 2:**
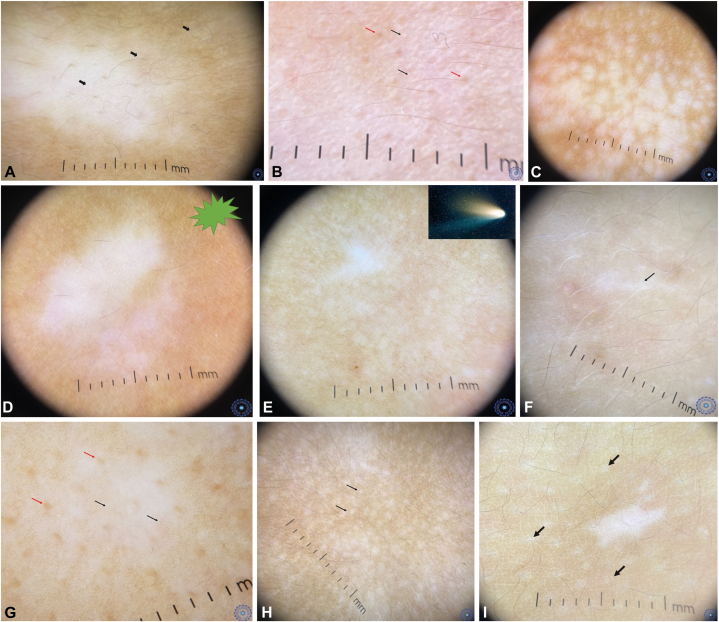
**A,** Progressive vitiligo lesion showing trichrome pattern (*black arrows* exposing 3 different colors). **B,** Progressive vitiligo lesion showing a reverse network (*black arrows*) and perifollicular residual pigment (*red arrows*). **C,** Progressive vitiligo lesion showing *white* nacreous globules. **D,** Progressive vitiligo lesion showing a starburst pattern. **E,** Progressive vitiligo lesion showing a comet-tail pattern. **F,** Progressive vitiligo lesion showing a micro-Koebner phenomenon (*black arrow*). **G,** Progressive vitiligo lesion showing perifollicular residual pigment (*red arrows*) and attenuated pigment network (*black arrows*). **H,** Progressive vitiligo lesion showing polka dots (*black arrows*). **I,** Progressive vitiligo lesion showing perilesional perifollicular depigmentation (*black arrows*).
  • white nacreous globules (Fig 2, *C*), starburst patterns (Fig 2, *D*), and comet-tail streaks (Fig 2, *E*);
  • micro-Koebner phenomenon (Fig 2, *F*), dotted vessels, and residual perifollicular pigmentation with leukotrichia (Fig 2, *G*);
  • perilesional abnormalities such as polka-dot patterns (Fig 2, *H*), perifollicular depigmentation (Fig 2, *I*), and peripheral reverse network (*P* < .01).
• *Stable lesions* were characterized by
  • the absence of progression signs and *sharp borders* (*P* < .01).
• *Repigmenting lesions* were associated with
  • perifollicular or border hyperpigmentation (Fig 3, *A*), intralesional telangiectasias or erythema (Fig 3, *B*), and perilesional erythema (Fig 3, *C*) (*P* < .01).

• *Progressing lesions* were significantly associated with
  • trichrome patterns (Fig 2, *A*), reverse network (Fig 2, *B*);

**Fig 3:**
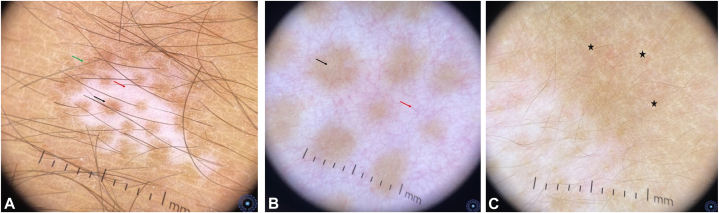
**A,** Repigmenting vitiligo lesion with a hyperpigmented border (*green arrow*), perifollicular hyperpigmentation (*black arrow*) and dotted vessels (*red arrow*). **B,** Repigmenting vitiligo lesion showing intralesional erythema and telangiectasias (*red arrow*) and perifollicular hyperpigmentation (*black arrow*). **C,** Repigmenting vitiligo lesion showing perilesional erythema (*black stars*).
• *Recent onset lesions* were distinguished by
  • attenuated network (Fig 4, *A*), perifollicular depigmentation, blurred borders (Fig 4, *B*), and perilesional leukotrichia (Fig 4, *C*) (*P* < .01).

**Fig 4:**
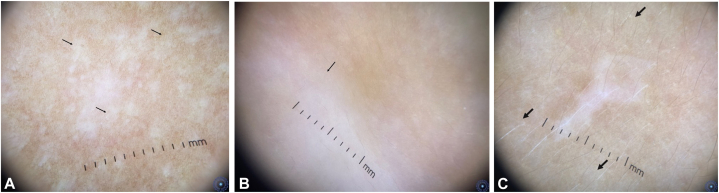
**A,** Recent onset vitiligo lesion showing lesional and perilesional attenuated pigment network (*black arrows*). **B,** Recent onset vitiligo lesion with a blurred border (*black arrow*). **C,** Recent onset vitiligo lesion with perilesional leukotrichia (*black arrows*).

**Table III:** Key dermoscopic signs depending on the activity group

Activity group	Key dermoscopic signs	*P* value
Progressing	Trichrome, reverse network, white nacreous globules, starburst, comet-tail, dotted vessels, perifollicular depigmentation, leukotrichia, perilesional alterations	*P* < .01
Stable	Sharp borders, absence of progression signs	*P* < .01
Repigmenting	Proximally pigmented bicolor hair, Perifollicular and border hyperpigmentation, erythema, telangiectasias	*P* < .01
Recent onset	Distally pigmented bicolor hair, Blurred borders, attenuated network, perilesional leukotrichia	*P* < .01

**Fig 5:**
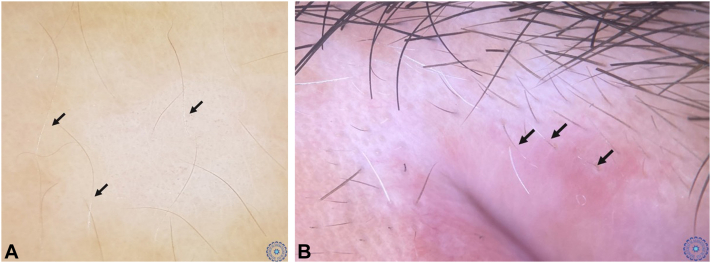
**A,** Recent onset vitiligo lesion displaying distally pigmented bicolor hairs (*black arrows*). **B,** Repigmenting vitiligo lesion showing proximally pigmented bicolor hairs (*black arrows*).
• *Proximally pigmented bicolor hair* (Fig 5, *B*), a new marker of repigmentation.

Two novel dermoscopic findings were identified:

• *Distally pigmented bicolor hair* (Fig 5, *A*), associated with recent lesions.

## Discussion

Vitiligo, an acquired leukoderma of multifactorial origin, involves melanocyte destruction through autoimmune mechanisms. Diagnosis relies on clinical examination and Wood's lamp findings,^15^ while management depends on disease activity and depigmentation extent.^9^ Reliable clinical markers of activity, such as the Koebner phenomenon, confetti depigmentation, trichrome pattern, inflammatory signs, and pruritus, help standardize assessments.^3^^,^^16^ Although scoring systems like the vitiligo extent score and the vitiligo area scoring index are approved,^17^^,^^18^ their practicality in routine practice is limited. Dermoscopy, therefore, emerges as a non-invasive, efficient alternative, especially in detecting subclinical features. Nevertheless, most studies describing dermoscopic findings have been descriptive, with no statistical correlation to reported results,^5^ highlighting the need for standardized assessments and proper training to minimize interobserver variability.^19^

Since the first report of Zawar et al’ in 2004,^4^ follicular opening changes have been recognized as key vitiligo-specific dermoscopic signs. Persistent perifollicular pigmentation, signifying residual melanocytes, is a favorable prognostic indicator for repigmentation.^4^^,^^13^ However, inconsistencies in dermoscopic nomenclature across studies highlight the need for standardized terminology.^6^^,^^20^

In our study, statistically significant dermoscopic markers of progression include starburst appearance, comet tail, micro-Koebner phenomenon, and peripheral white nacreous globules (Fig 2, *A*-*I*). Patterns like reverse network and perifollicular depigmentation further suggest disease activity.^13^^,^^21^ Notably, these findings emphasize the importance of assessing the entire lesion, not just its central areas.

Perifollicular pigmentation is variably interpreted: some associate it to repigmentation,^4^ while others consider it a marker of active disease.^10^^,^^13^ This discrepancy likely stems from the lack of distinction between perifollicular pigment retention and perifollicular repigmentation.^6^

Our findings support that perifollicular residual pigment was observed in progressing lesions, whereas perifollicular hyperpigmentation, characterized by a homogenous pigmentation or pigmented network >1 mm, was associated with repigmentation (Fig 3, *A*).

To assess lesion stability, Nirmal et al proposed a scoring system incorporating 6 dermoscopic signs.^12^ Our results support their observations regarding sharp borders and an absent pigment network indicating stability, while micro-Koebner and satellite lesions suggested disease activity.

Additionally, our study suggests that telangiectasias and erythema are significant markers of repigmenting lesions, in line with Awal et al^22^ (Fig 3, *B* and *C*).

Recent onset lesions evolving for less than 3 months were identified using photograph-based comparative analysis.^23^ These lesions exhibited an attenuated and ill-defined network (Fig 4, *B*) and perilesional leukotrichia (Fig 4, *C*), consistent with findings from Thatte and Khopkar on 30 vitiligo lesions evolving for less than 2 months.^10^

Anbar et al demonstrated that vitiliginous hair follicles contain both active and quiescent melanocytes, with functional melanocytes declining as lesion duration increases.^24^ This suggests that older lesions have a reduced capacity for spontaneous repigmentation, while a reservoir of dormant melanocytes persists in short-duration lesions within the follicular bulge.

Our novel follicular findings highlight the role of hairs in repigmentation and vitiligo. Distally pigmented bicolor hairs (Fig 5, *A*) were linked to recent lesions, possibly indicating an early loss of functional melanocytes, whereas proximally pigmented bicolor hairs (Fig 5, *B*) were associated with ongoing repigmentation, supporting the hypothesis of melanocyte migration from the follicular reservoir to the epidermis.

Finally, the dermoscopic analysis chart proposed herein offers a comprehensive yet practical approach for assessing vitiligo activity in daily practice.

### Limitations and perspectives

Most participants had received topical treatments or phototherapy, which may have influenced dermoscopic findings such as intralesional erythema and telangiectasia.^6^ This potential treatment bias should be considered when interpreting results. Future studies should stratify untreated versus treated patients to better assess the utility of dermoscopy in monitoring repigmentation and treatment response over time.^11^

Additionally, while our findings suggest a link between bicolor hairs and repigmentation dynamics, their histological correlate remains unclear. Further research integrating histopathological and immunohistochemical analyses could clarify whether bicolor hairs serve as an early marker of treatment-induced repigmentation and help refine dermoscopic assessment in vitiligo management. Consequently, larger and independent cohorts are needed to strengthen and validate dermoscopic findings in vitiligo assessment.

## Conclusion

This study corroborates previously published findings while offering additional insights through the analysis of a large dermoscopic dataset. A systematic dermoscopic assessment of vitiligo lesions—center, border, and periphery—can maximize diagnostic and prognostic value.

Key markers of activity include the trichrome pattern, reverse network, white nacreous globules, starburst, comet-tail pattern, micro-Koebner phenomenon, perifollicular residual pigmentation, leukotrichia, and polka dots. Stable lesions are distinguished by sharp borders.

Repigmentation indicators involve perifollicular and border hyperpigmentation, erythema, telangiectasias, and proximally pigmented bicolored hairs.

Early vitiligo lesions are characterized by an attenuated network, perifollicular depigmentation, blurred borders, perilesional leukotrichia, and distally pigmented bicolored hairs.

To ensure comparability across studies and improve clinical utility, standardizing dermoscopic nomenclature remains essential for future large-scale research.

## Conflicts of interest

None disclosed.
